# Exit strategies from lockdowns due to COVID-19: a scoping review

**DOI:** 10.1186/s12889-022-12845-2

**Published:** 2022-03-12

**Authors:** Madhavi Misra, Harsha Joshi, Rakesh Sarwal, Krishna D. Rao

**Affiliations:** 1 Johns Hopkins India Private Ltd, Flat 57, India International Centre, 40, Max Muller Marg, New Delhi, 110003 India; 2 National Institution for Transforming India Aayog, Delhi, India; 3 Johns Hopkins School of Public Health, Johns Hopkins University, Baltimore, USA

**Keywords:** COVID-19, Lockdown, Restriction, Exit strategy, Opening up, Exit plan, Pandemic

## Abstract

**Introduction:**

In response to the ongoing COVID-19 pandemic, countries have adopted various degrees of restrictive measures on people to reduce COVID-19 transmission. These measures have had significant social and economic costs. In the absence of therapeutics, and low vaccination coverage, strategies for a safe exit plan from a lockdown are required to mitigate the transmission and simultaneously re-open societies. Most countries have outlined or have implemented lockdown exit plans. The objective of this scoping review is to (a) identify and map the different strategies for exit from lockdowns, (b) document the effects of these exit strategies, and (c) discuss features of successful exit strategies based on the evidence.

**Methods:**

A five-step approach was used in this scoping review: (a) identifying the research question and inclusion/exclusion criteria; (b) searching the literature using keywords within PubMed and WHO databases; (c) study selection; (d) data extraction; (e) collating results and qualitative synthesis of findings.

**Results:**

Of the 406 unique studies found, 107 were kept for full-text review. Studies suggest the post-peak period as optimal timing for an exit, supplemented by other triggers such as sufficient health system capacity, and increased testing rate. A controlled and step-wise exit plan which is flexible and guided by information from surveillance systems is optimal. Studies recommend continued use of non-pharmaceutical interventions such as physical distancing, use of facemasks, and hygiene measures, in different combinations when exiting from a lockdown, even after optimal vaccination coverage has been attained.

**Conclusion:**

Reviewed studies have suggested adopting a multi-pronged strategy consisting of different approaches depending on the context. Among the different exit strategies reviewed (phase-wise exit, hard exit, and constant cyclic patterns of lockdown), phase-wise exit appears to be the optimal exit strategy.

**Supplementary Information:**

The online version contains supplementary material available at 10.1186/s12889-022-12845-2.

## Background

The ongoing pandemic of coronavirus disease 2019 (COVID-19) caused by the severe acute respiratory syndrome coronavirus 2 (SARS-CoV-2) has been responsible for infecting 362 million people. Around 5.6 million people have lost their lives [1]. To mitigate the effects of this pandemic, most countries have implemented various degrees of population movement restrictions. This has involved closing borders, closing non-essential workplaces and schools, restrictions on gatherings and movements of people (road, air, sea). Some of the mitigation strategies ranged from complete lockdown as seen in India [2] to moderate strategies like in the United Kingdom supported by increased testing, tracing, and quarantining [3]. Other mitigation measures included age-selective distancing. For instance, in New Zealand and South Africa, the older population and those with co-morbidities were recommended to be isolated at home, while the younger population were allowed to go to work [4].

These restrictive measures have significant social and economic consequences, especially in low income, and low- and middle-income countries (LMIC). These measures adversely affected the disadvantaged population as it led to the shutdown of economic activities, loss of employment, disruption in education, challenges in access to essential health services and other public services, including food insecurity [5]. There is, therefore, a need to exit from lockdowns while simultaneously mitigating the COVID-19 transmission. In the absence of therapeutics and a significant vaccination coverage, a situation that is commonly seen in many LMICs, there is a need for strategies for a safe exit from the restriction measures.

To circumvent the challenges faced following the lockdowns, countries have attempted to devise optimal strategies to exit from lockdowns. Many countries have defined graded exit plans with each phase informed by triggers such as case numbers, infection rate, health system capacity, etc. Other countries have relied on seroprevalence studies and increased testing prior to opening up. The phase-wise exit plans are usually structured by type of business, school, and size of gatherings, etc. The timing and containment measures during exit also varied. Almost all exit strategies studied suggest the continued use of non-pharmaceutical interventions (NPIs- These include physical distancing, use of face mask, and hygiene measures, and other restrictive measures such as stay at home, school closures, travel restrictions, border closures, and steps to address ventilation measures especially in closed spaces) in various combinations suited to country contexts. Countries such as New Zealand and South Korea began relaxing restrictions only after the number of new daily cases reached almost zero. Austria began implementing its exit plan when the daily caseload fell below 100, so the health system wasn’t overwhelmed [4]. However, many countries have opted to open up when transmission rates were falling, but had significant daily case numbers. Therefore, measures for containing the spread of disease need to be in place while opening, to avoid the health system from being overwhelmed by another wave.

The objective of this scoping review is to systematically document the evidence regarding exit strategies related to COVID-19 lockdowns. This scoping review has the following specific aims: (a) to identify and map the different strategies that have been adopted by countries, and are suggested from modelled scenarios of exit from COVID-19 related lockdowns, (b) document the effects of these exit strategies, and (c) discuss features of successful exit strategies based on the available evidence.

Countries are at different stages of the COVID-19 pandemic. While increasing vaccination coverage is a global goal, many countries have not been able to achieve this due to constraints related to vaccine availability and affordability. With new variants emerging such as Omicron in November 2021, some countries responded with a knee-jerk reaction of imposing travel bans and strict lockdowns [6, 7]. The World Health Organization (WHO) has criticized travel bans, as they affect lives and livelihoods more than the spread of virus [8]. Planning how best to exit from lockdowns is an important policy and public health decision. Depending on the local health system capacity, ongoing calibration of restriction measures is required. There is limited evidence so far on the effects of different exit strategies and findings from this scoping review can guide countries in identifying optimal exit strategies.

## Methods

### Search strategy and selection criteria

This scoping review was based on searches conducted on the PubMed (https://pubmed.ncbi.nlm.nih.gov) and WHO (https://www.who.int) databases in the interest of time (Electronic search strategy for PubMed database provided as Additional file 1). The inclusion criteria for the search included studies from all countries and the time frame was the start of the pandemic in 2020 till May 2021 (refer to Table 1 on PICOS framework). The most recent electronic database search was conducted on 7th June 2021. Full-length, peer-reviewed and pre-print literature available in the English language related to exiting from a lockdown/opening from a lockdown/ removal of lockdowns was included. All clinical studies including drug trials, hospital-based studies, and vaccine efficacy studies were excluded.

**Table 1:** Inclusion and Exclusion criteria (PICOS framework)

Criteria	Inclusion	Exclusion
**Population**	All countries	None
**Intervention**	Effectiveness of exit strategies on COVID-19 outcome, effectiveness of vaccination in relation to opening up/lockdown strategies	All clinical, hospital-based studies, drug trials, effectiveness of strategies on non-COVID 19 outcomes, vaccine effectiveness
**Comparator**	None	None
**Outcome**	COVID-19 incidence/prevalence, transmission factor	Non-COVID 19 outcomes
**Study design**	Observational studies, modelling studies, reviews	Randomized controlled trials, opinion editorials, commentaries, and letters to the editor

### Data synthesis

After removing the duplicates, two reviewers (MM and HJ) independently examined the abstracts and selected 197 articles for full-text review. Ninety articles were removed as they did not match inclusion criteria as per PICOS and 107 articles were retained for the review. The exclusions after full-text review were discussed among the two reviewers. Data from the included articles were extracted in an excel sheet under pre-populated themes on the timing of exit, determinants for exit, process of exit, components of exit strategy, and effects of opening up. Qualitative synthesis of findings was undertaken and reviewed by all four authors (MM, HJ, RS and KR).

## Results

### Search outcomes

**Fig. 1:**
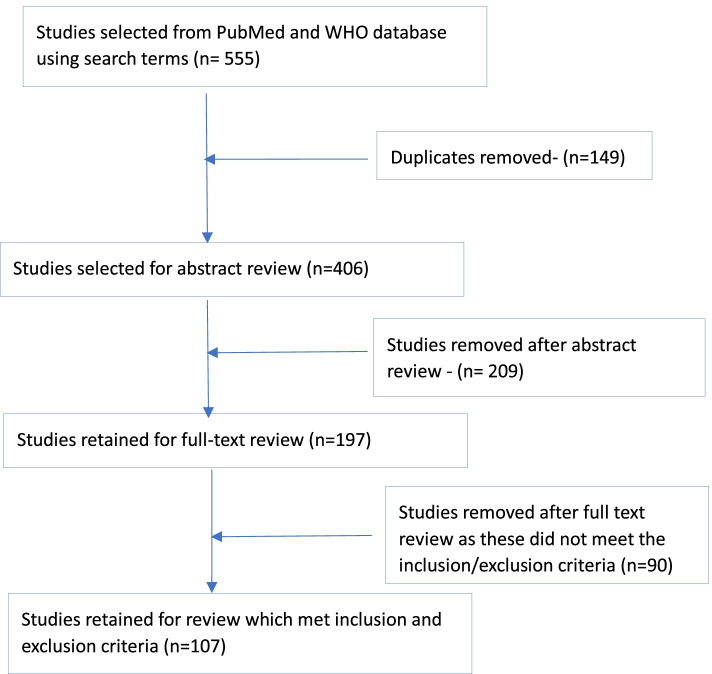
Study selection flow chart

The search yielded 555 articles using keywords such as Covid, non-pharmaceutical interventions (NPIs), exiting from lockdown, and exit strategies. We found 406 unique studies combining both databases and after reviewing the abstracts and full-text review, retained 107 studies as a part of this review (refer to Fig. 1 on study selection flow chart).

### Description of the included studies

**Fig. 2:**
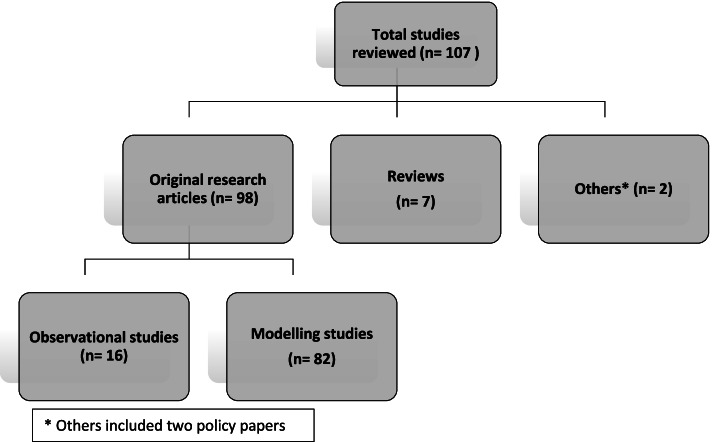
Types of studies included in the review

Of the 107 studies, 98 (91.5%) were original research studies, seven (6.5%) were reviews and two (2%) were policy papers. Of the 98 original research studies, 82 studies (84%) were based on mathematical modelling, and 16 (16%) were observational studies (refer to Fig. 2 on types of studies found).

The majority of the studies (68%) were from high-income countries (as per the World Bank classification of countries by income, accessed from https://datahelpdesk.worldbank.org/knowledgebase/articles/906519-world-bank-country-and-lending-groups), about 18% of studies were based in middle-income countries and only two studies were based in low-income countries. The review found eleven studies (11%) that had used global databases.

### Types of exit strategies and their effects

Based on the review, we have identified the following key themes to describe an exit strategy (refer to Table 2).

1.  Timing of exit- Here the focus is on pre-requisites for opening up after lockdown and determinants for deciding the timing of exit.
2.  Process of exit- This section covers strategies adopted to exit from lockdown and the effect they have had on COVID-19 outcomes.
3. Supporting conditions for exit strategies- This section reports on the types of public health measures for containment, and use of NPIs during exit from lockdown.

**Table 2:** Theme-wise summary of reviewed studies

Sr. No.	Key themes	Number of papers addressing the theme^a^	Percentage of papers addressing the theme
1	Timing of exit strategy	41	38.3
2	Process of exit	36	33.6
3	Supporting conditions for exit strategy	64	68.4

^a^ Some papers address more than one theme; thus, the total number exceeds 107

#### 1. Timing of the exit

##### Post peak period- reducing number of cases and better health system capacity

Two review articles based on global data [9, 10] observe that most countries have opted for opening during the ‘post-peak period’. In this period, a plateauing of cases and hospital admissions are maintained for 2 weeks, implying that the health system can cater to the cases without a crisis. However, this approach risks the formation of new clusters, triggering the next wave of the pandemic, as most of the population is likely not exposed yet to the virus. A strong surveillance system to classify the epidemiological situation is required during the post-peak period. Petersen et al. [10] have adopted the WHO guideline to classify new cases in the post-peak period as imported (from other countries), part of a known cluster, or those with an unknown source.

An article [9] has highlighted that governments need to take into account individual healthcare, economic and social considerations while deciding on the timing of opening, emphasizing that context is important. A policy paper [4] has summarized various triggers to determine the timing for exiting from lockdowns such as (a) health system capacity (number of beds, equipment available), (b) health system demand (e.g. ICU admissions), (c) death rate (e.g. New York’s plan to ease restrictions after 14 days continuous fall in death rate), (d) mode of transmission (New Zealand’s plan explicitly relates easing levels with change in the mode of transmission- to households only), and (e) cost-benefit analysis. Cuschieri [11] has described Malta’s experience of reopening from the COVID-19 lockdown where the government considered gradual relaxation of lockdown when the ‘Reproduction number’ (R- The effective reproductive number is the average number of secondary cases per infectious case in a population made up of both susceptible and non-susceptible hosts. If R > 1, the number of cases will increase, such as at the start of an epidemic, and where R < 1 there will be a decline in the number of cases [12])’ went below one. Raje et al. [13] have found crossover time point (when the case recovery rate is greater than case active rate) as an effective trigger to initiate relaxation of restrictions, based on country experiences. Importance of an evidence-based approach for the timing of exit, which takes into account prevalence and spread of the disease has been advocated in several studies [14, 15].

Findings from modelling studies are corroborated by empirical studies (refer to Table 3). These studies demonstrate the postponing of restrictions, farther beyond the peak may have additional benefits in reducing the number of cases. A US based [16] and an India-based [17] modelling study shows benefits of prolonged removal of restrictions possibly due to progressive exhaustion of the infectious pool in the population.

**Table 3:** Timing of the exit- Findings from the epidemiological modelling studies

Sr. No.	Country	Study	Determinants of opening-up	Effect on timing for opening-up
1	USA	Zhang et al. [16]	Peak in number of COVID-19 cases, Current state of the infectious population, and the remaining susceptible population (estimated using epi models)	• Prolonged removal of restrictions in the post-peak period has benefits • Delay in reopening by one month can lead to an average reduction of new cases by 42%.
2	India	Gupta et al. [17]	Peak in number of COVID-19 cases	• Delaying the reopening farther beyond the peak has benefits due to progressive exhaustion of infectious pool in the population
3	Italy	Scala et al. [18]	Peak in number of COVID-19 cases, Strength of lockdown, Geography	• Premature exit before the peak can result in the next wave with a higher peak. • Increasing the strength of the lockdown can delay the time for opening • Epidemic dynamics vary between regions and are independent of each other, therefore, lockdown lifting time is to be evaluated regionally.
4	Global	Roy [19]	Peak in number of COVID-19 cases and health system capacity	• Premature exit following a brief reduction in cases can result in quicker, sharper, and higher secondary peak • Continuing lockdown till the peak reduces to health system capacity level can lead to a secondary peak which is above the health system capacity • Reopening after the cases have plateaued, and are well below the health system capacity will lead to a much lower secondary peak.
5	UK	Nekovee [20]	Peak in number of COVID-19 cases	Premature lifting of mobility restrictions can result in the return of COVID-19’s exponential growth
6	Italy	Li et al. [21]	True number of infected cases and relative testing capacity	Local testing capacity should be more than 16 times the estimated true number of newly infected cases for opening-up
7	UK	Moore et al. [22]	Vaccine efficacy, vaccine uptake	• Early relaxation of NPIs before sufficient immunity has been achieved can lead to a larger wave of infection • If all restrictions are removed only after the entire adult population has been offered two doses (assuming vaccine provides 85% protection against infection), there will still be a next infection wave. (Except, when vaccine uptake is 95, 90, and 85% in those aged 80 years and older, 50–79 years, and 18–49 years, respectively)

Findings from an Italy-based modelling study [18] suggest that the stricter the period of lockdown, the longer it might take to exit from the lockdown. In this scenario of a strict lockdown, the subsequent wave is anticipated to be stronger as well. Similarly, if the lockdown is lifted before reaching the peak of the COVID-19 cases, the next wave of cases will have a sharper peak. Studies based in different contexts [18–20] have modelled scenarios of premature exit and demonstrated the risk of sharper and rapid infection peaks.

Another modelling study from Italy [21] hypothesized that the true number of infected cases and relative testing capacity are better determinants to guide lockdown exit strategies. It concludes that decisions on opening should be taken at the local/regional level based on capacity to identify new cases and social contacts. Based on a modelling exercise on data from different regions of Italy, local testing capacity was suggested to be more than 16 times the estimated true number of newly infected cases if a decision to re-open is to be taken. Sufficient health system capacity to cater to stable daily cases was emphasized as a determining factor for opening up [23].

##### Vaccination coverage/immunity

Vaccines to protect from COVID-19 are now an important part of the exit strategy. Moore et al. [22] estimate the effects of vaccination coverage and lifting of restrictions in the UK-based modelling study. The study finds future waves of infection and deaths can be reduced by increasing levels of vaccine-derived immunity in the population. Early relaxation of NPIs before sufficient immunity has been achieved can lead to a larger wave of infection. Furthermore, the study adds vaccination alone cannot bring R below one, and control the epidemic. As per the modelling exercises, with assumptions of vaccine offering 85% protection against the infection and vaccine uptake above 75%, R would reduce to 1.58, which is still greater than the required value of below one. Therefore, NPIs such as face masks, physical distancing, and hygiene measures are required, even after the adult population is fully vaccinated and a stricter lockdown has been lifted.

#### 2. Process of exit

Thirty-six studies in the review examined different processes of exit- a) phase-wise/progressive/gradual, b) hard exit (resuming all activities at one time), c) cyclic exit (short cycles of opening and closing) and, d) zonal lockdowns (containment in clusters). The majority of studies have identified phase-wise exit as the most appropriate strategy (refer to Table 4).

**Table 4:** Summary of studies examining processes of exit

Sr. No.	Type of exit process	No. of papers (*n* = 36)	Percentage of papers
1	Phase-wise/gradual	28	77.8%
2	Cyclic	5	13.9%
3	Zonal	3	8.3%

Six studies [24–29] included comparisons of different exit strategies. Out of these, four studies [24, 26, 28, 29] compared hard exit with gradual exit and concluded gradual exit to be effective. One study [25] compared zonal strategy with cyclic strategy and concluded zonal strategy to be effective in LMIC settings. The sixth study [27] was inconclusive about the findings.

Findings related to the process of exit from the select studies have been listed in Table 5.

**Table 5:** Process of exiting- Findings on types and effects of suggested exit strategies from the reviewed studies

1. Phase wise opening up				
**Country**	**Study (type of study)**	**Details of strategy**	**Determinants**	**Effects**
Belgium	Abrams et al. [30] (Modelling study)	Phase 1b —Shops re-opened under strict requirements related to the organization of the work and restricting access to the store to avoid overcrowding; Phase 2a — Schools partially re-opened (first phase —selected grades in primary and secondary schools); Phase 2b — Schools partially re-opened further (second phase — pre-primary schools); Phase 3 — Restaurants, bars, and cafes re-opened un-der strict measures including physical distancing and a limited number of customers;	Based on the daily number of new hospitalizations and admissions to the ICU.	None given
	Coletti et al. [31] (Modelling study)	Phase 1 – Work-places reopen Phase 2 – Schools reopen Phase 3 – Leisure activities reopen	Regular re-assessment is crucial to adjust to evolving behavioural changes that can affect epidemic diffusion. In addition to social distancing, sufficient capacity for extensive testing and contact tracing is essential for successful mitigation.	None given
Germany	Dorn et al. [32] (Modelling study)	Gradual lifting of shut down	Long duration of remaining restrictions would increase relative economic costs compared to alternative gradual opening strategies	Reproduction number is around 0.8.
USA	Gulbudak et al. [33] (Modelling study)	Rapid measured lockdown with intermediate fatigue (rapid reactive lockdown as soon as possible) in conjunction with the subsequent wave being detected lasting 30 days before 50% return to normalcy	Sustained public social distancing and mask wearing, targeting transmission reduction rather than removing susceptible altogether, to reduce R.	None given
India	Bhattacharya et al. [34] (Modelling study)	Graded/staggard exit Progressive social awareness		This can minimize the peak and flatten the infection curve.
	Goel et al. [35] (Observational study)	Phase 1-Relaxation of all zones except containment zones. Opening of liquor shops. Govt offices opened with 33% capacity. Movement with a pass. Phase 2 - Domestic travel resumes. Opening commercial activity decided at the state level. Phase 3- Lockdown in containment zones and social gatherings and venues closed. Phase 4 – Night-time curfew from 9 pm-5 am. Phase 5 - Gyms and yoga institutes open. Revocation of night curfew.	Economic relief measures Technological advances Evolution of testing criteria and testing methods Strengthening health system	Any initial success of handling the pandemic will not last without continuous and reliable testing followed by contact tracing.
**2. Cyclic/ rolling lockdown**				
France	Boulmezaoud et al. [36] (Modelling study)	Zigzag strategy of alternating between periodic and moderate deconfinement. The period should remain small compared to the time needed to reach the peak of the epidemic if deconfinement is maintained (which is in the order of 4 to 5 months). A periodic deconfinement is equivalent to a weekly organized deconfinement with 3 and a half days of strict lockdown per week. Scenarios alternating strict lockdown and moderate deconfinement can allow the epidemic to be brought under control without resorting to group immunity.		Moderate deconfinement with strong but non-drastic interventions, whether gradual or sudden, can lead to a rapid resumption of the epidemic, with a saturation of intensive care units in the fall and a peak of the epidemic in winter.
Germany	German et al. [37] (Modelling study)	Repetitive short-term contact reductions. Such reductions can be triggered adaptively if death rates, need for ICU, etc. exceed a threshold. With additional hygienic measures, the situation can be enhanced further. However, repetitive short-term lockdowns and hygiene measures need to be in place for the next two or three years until herd immunity can be obtained (if vaccination is not available before).	The effects of antibody tests would add significant benefit to exclude people with antibodies from the contact reductions.	None given
**3. Zonal lockdown**				
India	Chowdhury et al. [25] (Policy paper- overview)	Zonal or local lockdowns may be suitable for some countries where systematic identification of new outbreak clusters in real-time would be feasible	Requires generalised testing and surveillance structure, and a well-thought-out (and executed) zone management plan.	None given

##### Phase-wise

Studies done in Belgium [30, 31, 38], Germany [32], US [33], Netherlands [39], Spain [40], and India [34, 35] show evidence for a phase-wise opening. A study from Germany [41] suggests phase-wise opening should be reversible (i.e., if the reproduction number- R starts going up, the lockdowns can be easily re-imposed) and be pilot tested for four-eight weeks before complete opening up of all restrictions.

##### Cyclic/rolling lockdown

- To get to an optimal exit strategy, a study from Germany [37] suggests repetitive or rolling lockdowns for up to two-three years by which time herd immunity is reached as this would keep the R under one. This study suggests policymakers must weigh the extent of restrictions against the economic consequences. The conflict between health protection and economic interests needs reconciliation while opening from the lockdown.
- Another modelling study from France [36] proposes a cyclic or a zig-zag schedule of four-day work and 10-day lockdown which can prevent a resurgence and also provide part-time employment. This strategy suggests a drastic, cautionary, or a relaxed approach to lockdowns that must be supported by strict implementation of NPIs (hand hygiene, face mask use, physical distancing, and testing, contact tracing, and quarantine).
- These studies suggest considering economic and social costs before implementing a cyclic or rolling lockdown which has logistic challenges. They caution that periodic lockdown and openings do not lead to herd immunity. Transitioning from one phase to the next is made after measuring the impact of deconfinement by estimating the daily R.

##### Zonal lockdown [25]

- Zonal lockdowns are local lockdowns where specific ‘hotspots’ have a sudden outbreak cluster (high number of cases) which have been identified in real-time. Such clustered social distancing works by dividing the population into “zones” according to the geospatial distribution. The disease clusters are contained within these zones so that interactions within a zone are significantly greater than interactions between them. An India-based study [42] suggests that containment zones with a higher case-load should remain even during the exit phase.
- Although effective in developed countries, a study based in LMICs [25] suggests zonal lockdowns with the relaxation of restrictions in remaining places has challenges in LMIC, due to the absence of large-scale population surveillance system and limited testing facilities.

A modelling study [28] compared the effectiveness of different types of exit strategies- hard exit, progressive exit, and cyclic exit (2 weeks of lockdown and 2 weeks of opening over four cycles) and maintaining status quo. Evolution of the *Rt* (effective reproduction number at a particular time is the expected number of new infections caused by an infectious individual in a population where some individuals may no longer be susceptible [12]) values for the four exit strategies modelled for Luxembourg, Italy, and Japan found that progressive exit offered better outcomes in terms of little impact on the economy and reduced number of cases. A Singapore-based study [43] concludes the effectiveness of gradual relaxation in flattening the curve compared to a sudden resumption of social interactions.

#### 3. Supporting conditions for exit strategies

Steps supporting the exit strategy and facilitating its successful implementation include public health (these include testing, contact tracing, quarantine and isolation, and surveillance), pharmaceutical (such as treatment, drug therapies, and vaccination), and non-pharmaceutical interventions (NPIs- these include physical distancing, use of face masks, and hygiene measures). Other restrictive NPIs such as stay at home, school closures, travel restrictions, border closures, and steps to address ventilation measures especially in closed spaces are implemented in varying degrees depending upon the number of cases). This review is focused on public health and NPIs as measures of exiting from a lockdown.

##### Health system and public health capacity

Along with ensuring physical distancing and reducing contacts to control the transmission, the purpose, and justification for lockdowns have been to strengthen the capacity of health systems. This would include not only the facility level capacity, but also the public health capacity in terms of testing, tracing, quarantine and isolation.

- Testing and surveillance

In the studies reviewed [10, 17, 24, 44–52], upscaling the antigen testing capacity is identified as a critical requirement while planning for exiting the lockdown. Countries that implemented testing at an early stage along with tracing and quarantine could effectively control the spread of COVID-19. For example, South Korea relied on ‘trace, test and treat’ strategy to control the epidemic without imposing nationwide lockdown [53]. This implies the need for extensive testing capacity before considering reopening (refer to Table 6).

**Table 6:** Testing strategies to support exiting from lockdown: Findings from modelling studies

Sr. No.	Country	Study	Testing strategy	Effect of testing strategy to support existing from lockdown
1	Switzerland	Muller et al. [45]	Daily random testing	• Daily random testing will reduce the delay between changes in policy and the observation of their effects • Additional testing capacity of 15,000 per day carried out randomly would provide data about the evolution of the epidemic during exit.
2	UK	Panovska-Griffith et al. [46]	Active testing of symptomatic population	Increased levels of testing (between 59 and 87% of symptomatic people tested at some point during an active COVID-19 infection) and effective contact tracing and isolation for infected individuals can prevent rebound of the epidemic during reopening of schools and society in UK.
3	Mendoza, Argentina	Mayorga et al. [47]	Extensive testing capacity to detect asymptomatic individuals	Massive COVID-19 screening to detect around half of the asymptomatic and very mildly affected individuals would not need strict suppressive actions- if 45% of asymptomatic individuals are detected through testing and are isolated, there would not be a need for lockdown. (This modelling exercise was undertaken with assumptions- a) imposing lockdown when ICU beds occupancy reaches 50%, and b) relaxing restrictions when this value reaches 30%)
4	India	Gupta et al. [17]	Increased testing	Lower restrictive measures along with increased testing during lockdown relaxation have the same effect as stricter physical distancing measures with lower levels of testing.
5	Italy	Li et al. [21]	Upscaling the testing capacity	• True number of infected cases and relative testing capacity are better determinants to guide lockdown exit strategies, compared to R. • Testing capacity of at least 16 times the number of newly infected cases is required before considering exit at regional levels in Italy.
6	Australia	Lokuge et al. [54]	Community-based surveillance strategy using pooling of samples	• Exhaustive testing of patients with respiratory symptoms in the community is the most efficient and feasible means of detecting community transmission of COVID-19 during relaxation of measures. • Pooling allows increased case detection when testing capacity is limited, even given reduced test sensitivity.
7	Italy	Pernice et al. [55]	Targeted testing in high-risk groups and contact tracing	• Contact tracing and targeted testing in high-risk groups would provide the same result as larger number of untargeted (or less targeted) tests. • Targeted testing approach is more efficient and feasible.
8	NA	Bej et al. [56]	Pro-active testing (testing beyond those who show symptoms)	• Compared effects of different exit strategies with high/low levels of pro-active testing. Strategies that lack high levels of pro-active testing led to a second wave of infection.
9	USA	Tam et al. [57]	Expanding testing capacity and encouraging early testing	• Infection rate can be decreased by increasing the sum of testing rate and recovery rate of asymptomatic individuals, after lifting the stay-at-home orders.

Review articles [10, 24, 48, 49, 51] included in this scoping review have emphasized the need for greater access to testing to allow the identification of new cases and clusters as early as possible. Massive testing of the healthy and infected population would be essential to inform policymakers about the effect of interventions during reopening.

Modelling studies included in the review [17, 21, 45–47, 54–57] have identified different effective testing strategies (listed in Table 6) to support opening, post lockdown.

Randazzo et al. [58] used wastewater surveillance and wastewater-based epidemiology to estimate the presence and prevalence of COVID-19 in communities. Findings suggest environmental surveillance could be implemented by municipalities as a tool for mapping high-risk areas during exit. Digital technology has been suggested for large-scale surveillance [59] and monitoring of epidemic [60] to support exit strategies in the reviewed studies.

b) Contact tracing, quarantine, and isolation

Improved health systems and public health capacity for contact tracing and ensuring quarantine and isolation are identified as prerequisites for opening up in the reviewed studies. This was necessary to identify and contain emerging clusters [26, 27, 61–64].

Kretzschmar et al. [61] have examined different scenarios of isolation and contact tracing settings in combination with social distancing levels for a safe exit strategy. Their modelling study results emphasize tracing non-household contacts during relaxation of restrictions. If not feasible due to public health system constraints, tracing and isolation of only household contacts is also found to significantly reduce the doubling time of the epidemic. A US-based modelling study [62] finds that increasing the capacity for detection, contact tracing and quarantine by at-least two folds would control the cases from rising during medium risk reopening (effective contact rate increased by three–five folds was considered as medium risk opening in the study).

Contact tracing using digital technologies has been suggested in some of the reviewed studies [24, 51, 65]. The acceptability of tracing apps has shown mixed results in this review. A study in Germany [66], found that people preferred to avoid mandatory tracing apps during exit strategy, while a cross-country study [67] from France, Germany, Italy, the UK, and the US found strong support for use of apps.

##### Non-pharmaceutical interventions

- Lifting restrictions on physical distancing

Ensuring strict physical distancing through lockdowns has helped to control the pandemic; however, this has had a profound ill effect on the economy. Reviewed studies examined various options for lifting physical distancing without increasing COVID-19 cases. We categorize these as below.

*Segmenting and shielding at-risk population*

Continuing the restrictions and ensuring physical distancing for at-risk population (those above 65 years of age, people living in care institutions, and those with chronic conditions) for an extended period compared to other individuals in society has been suggested in reviewed studies. For example, a UK-based modelling [68] study found that if restrictions are continued only for older (60+) and vulnerable people, there will be reduction in hospitalization by 50%, while if restrictions are continued for 50+ population with chronic diseases, the reduction will be by 57%. Few other studies based in the UK [69–71], France [26, 72], China [73], Pakistan [74], and Italy [75] have suggested a similar age-selective restriction strategy for opening up. A study from Brazil [76] refers to age-specific confinement as “vertical confinement”. This study finds “vertical confinement” would only be effective for all those over 50 years of age but this would then include the population in the working-age group and thus is not recommended.

A modelling paper [77] based in the UK suggests the strategy of segmenting and shielding the vulnerable. Dividing the population into groups that are relatively homogenous in healthcare needs is defined as segmenting. Those above the age of 70 years in receipt of government advice to shield/ in care homes/ receiving care at home are categorized as vulnerable. A study done in Nepal [44] recommends targeted closure and shielding of vulnerable and at-risk populations such as migrants, core case contacts, and family members. The exposure levels of household contacts/ contact with confirmed cases, exposure of border security forces, airport staff, health workers, and front-line workers should all be categorised in terms of high, medium, low, and no identifiable risk. This should be followed by active case management and monitoring based on asymptomatic and symptomatic cases.

The principle supporting the theory of protecting the vulnerable and allowing the healthy ones to carry out regular tasks assumes that it could help a majority of the population to return to normal. Risk classification tools to identify individuals who would require shielding during relaxation of interventions have been suggested in the studies [78, 79].

Although effective, this strategy may not be acceptable and feasible in all contexts. As observed in study findings from Brazil [76], such a strict age selective containment would not be possible in multi-generational households, especially in LMICs. Similarly, the implications of this strategy need to be interpreted along with considerations for its practical feasibility and potential wider benefits and drawbacks.

*Categorizing high-risk places*

From the studies reviewed, mapping of places with high transmission risk, super-spreading events, hotspots, and predicting mobility patterns is suggested before opening [80]. This information would help in designing policies to keep active surveillance of such places or to keep these areas closed while lifting the lockdown.

A review article [81] based on global data has identified indoor settings linked to increased risk of COVID-19 transmission. Large numbers of cases were from hospitals and elderly care settings in Europe. Other clusters with more than 100 cases included large religious gatherings, food processing plants, shopping places, and large cohabiting settings (worker dormitories, prisons, and ships). Settings with 50-100 cases included weddings, sports venues, bars, shopping places, and workplaces. Only a small number of clusters were related to schools and cases were most often reported among teachers and staff. An observational study [82] from eight high-income countries concurred with these findings.

Other studies [83–85] based in high-income countries have identified schools as low-risk settings with minimal effect on transmission after their opening compared to other indoor settings.

A UK-based modelling study [86] examined the effect of opening schools along with removing restrictions on different occupation groups and concluded increased transmission, i.e., R0 above one (the basic reproduction number, R0 is the average number of secondary infections produced by a typical case of an infection in a population where everyone is susceptible [12]). Continued restriction on recreational activities (e.g., restaurants and bars) during exiting has been suggested in a modelling study based in high-income countries [87].

*Daily contact rates*

Some modelling studies have suggested a combination of optimal daily contacts to bring R below one. A study based in the UK [88] suggests that while opening up, if the daily contact rate of an individual is maintained at five-six people, R would be reduced below one. For a contact rate of six-seven people, R may increase above one. For eight-nine people contact rate, health system capacity would be insufficient and may require another lockdown.

*Creating social bubbles*

A UK-based modelling study [89] examined the use of social bubbles or contact clustering to reduce contacts while opening from a lockdown. This means that two households would have exclusive contact and form a social bubble. Findings suggest that such a strategy can reduce COVID-related fatality by 42% as opposed to unrestricted socializing. The study shows epidemic risk can be further reduced if the transmission risk within the bubble is minimised.

In the reviewed studies, the importance of continuing some form of physical distancing during exit has been reiterated. E.g., one of the modelling studies [90] concludes sensitivity of the second wave to physical distancing rather than movements in the UK. Thus, indicating a need for physical distancing while opening up.

b) Use of Face Masks

The universal use of face masks after relaxing restrictions has been suggested as an effective exit strategy. Wearing masks by at least 60% of people was found to be a reasonable public health goal and at the same time a plausible strategy. It was found to be much easier than enforcing physical distancing in a modelling study in Australia [91]. This study found using only face masks reduced infections by 54% while using only physical distancing reduced infection by 24.7%. Especially in dense areas, face mask use is effective.

Another study [92] has shown that face mask use by infectious as well as the susceptible individuals is most effective as it reduces infection chances to 10% compared to 90% if none were wearing a mask. Wang et al. [93] conducted a modelling study using data from China, Italy, UK, and USA concluding the most effective exit strategy would be a combination of physical distancing and face mask use along with intense monitoring of the epidemic.

c) Relaxing travel restrictions

During opening up, a sustainable border control policy should be in sync with internal control measures. A modelling study [94] based on global data suggests opening of borders of countries and states where COVID-19 spread has already been successfully contained by internal measures. Pre-departure screening and testing on arrival are sufficient to keep imported cases in check without any border/travel restrictions [95]. Another modelling study [96] based in the EU correlates a mobility model to passenger air traffic and finds unconstrained mobility would have significantly accelerated the spreading of COVID-19. This was especially so in Central Europe, Spain, and France. Network epidemiology can inform political decision making and help countries exit from total lockdown.

## Discussion

Two years since the first outbreak of COVID-19, and after almost a year and a half of varying restrictive measures, countries had begun to open up from lockdowns in mid-2021. Countries that implemented moderate to severe measures to control the COVID-19 transmission faced challenges. In particular, to devise a safe exit plan, which would limit the transmission and have minimum social and economic costs. Some countries have been reimposing restrictions in light of increasing cases and emergence of newer variants in November-December 2021. Synthesis of the available evidence on exit strategies can help in policy decision making and analysis of this was found lacking.

This scoping review was undertaken to understand the different strategies that countries adopted to exit from lockdowns to mitigate the spread of COVID-19 and to document the effects of these exit strategies.

The majority of the studies adhering to the inclusion criteria were from the high-income countries (68%) and were based on epidemiological modelling exercises (76%), and therefore the results need to be interpreted with caution. We have described the exit strategies around the themes of timing, processes, and supporting conditions for exit answering key questions about when, how, and what.

We find the relaxation of restrictions is most appropriate when there is a decrease in the number of cases after the peak period for at least 2 weeks. This would prevent the health system from being overwhelmed. Determinants such as reduction in the reproduction number, a smaller susceptible population, considerations for the economy, livelihoods, and health system capacity are additional considerations before opening up. This corresponds to the WHO recommendation [97] to undertake a situational assessment of the intensity of transmission and health system capacity, before deciding to lift restrictive measures. WHO’s guidance on implementing and adjusting the public health and social measures, emphasizes the importance of flexible decision making for exiting at local levels, in coordination with neighbouring areas at the sub-national as well as the national level [97].

Most of the reviewed studies suggest phase-wise exit to be more effective compared to a hard exit or cyclic lockdowns, considering public health, clinical and social factors. WHO has repeatedly suggested [97, 98] slow, controlled and step-wise relaxation of measures. Moreover, an interval of 2 weeks has been recommended to identify any adverse effects of such measures and adjust the next steps accordingly.

This review suggests the importance of sufficient testing capacity and the need for extensive testing as necessary conditions while exiting. Almost all studies recommend continued use of non-pharmaceutical interventions in different combinations when exiting from a lockdown, which need to be in place even after optimal vaccination coverage has been attained. This is also in line with the WHO recommendation [97] for continuously monitoring the transmission levels and adopting appropriate public health measures, even when vaccination has begun. The studies reviewed also suggest the need for the maintenance of strong infection control measures in health establishments. For international travellers coming from countries with an active outbreak, strict quarantine rules should continue. Strengthening the public health system for detection, tracing, and quarantine should continue till vaccine coverage improves.

Adopting a multi-pronged strategy consisting of these different approaches as per the context is recommended by most studies we reviewed. In the high-income countries, there is a relatively larger proportion of formal sector workers and better health system capacity. Here, we note a reliance on increased testing capacity, and better surveillance to aid the phase-wise opening. In the low-and-middle-income countries, while the principles for opening up remain the same, studies have additionally suggested zonal lockdowns, local and context-specific identification of high-risk places and vulnerable individuals, and more adherence to non-pharmaceutical measures.

This scoping review has a few limitations. First, only two databases were included in the search strategy, likely missing out on other published evidence. However, we expect to have covered the majority of published studies. Second, the review has included studies on the effects of exit strategies on COVID-19 related outcomes. Effects on other outcomes such as social and economic aspects were not included. Effects of vaccination coverage on opening up weren’t studied as literature on this was in early stages while undertaking the review. Recent literature does point to significant effects of existing COVID vaccines on reducing disease severity (hospitalization and deaths) [99], and on newer variants such as Omicron [100]. Improving vaccination coverage thus becomes an important component of exit strategy. However, due to global vaccine divide, ‘vaccination’ may not be a feasible strategy for many countries at the moment. Further research needs to be undertaken to understand effects of vaccines on exiting from lockdowns in different contexts.

## Conclusion

Different approaches for exit strategies have been adopted by countries or suggested via modelling exercises in the review findings. These vary from imposing a phase-wise exit to a hard exit. Other strategies which consider vaccination coverage include constant partial lockdowns or a cyclic strategy for lockdown and relaxation till optimum immunity is achieved. Out of these, the phase-wise exit with continuation of non-pharmaceutical interventions appears to be optimal, as per the review findings.

## Supplementary Information

**Additional file 1.** Electronic search strategy for PubMed database.

## Data Availability

All data relevant to the study are included in the article or uploaded as supplementary information.

## Abbreviations

EU: European Union
NPI: Non-Pharmaceutical Interventions
WHO: World Health Organization
LMIC: Low- and Middle-Income countries
ICU: Intensive care Unit
