# Supplementary material for: Exit strategies from lockdowns due to COVID-19: a scoping review
Source: BMC Public Health. 2022 Mar 12;22:488. doi: 10.1186/s12889-022-12845-2 (PMC8917328; doi:10.1186/s12889-022-12845-2)
Supplement: Supplementary file 1 — Additional file 1. Electronic search strategy for PubMed database. [file 12889_2022_12845_MOESM1_ESM.docx]

**Electronic search strategy (All Searches run on June 7, 2021)**

**PubMed Searches**

((("COVID-19"[Mesh] OR "COVID-19"[all fields] OR "COVID 19"[all fields] OR "COVID19"[all fields] OR "COVID2019"[all fields] OR "COVID 2019"[all fields] OR "COVID-2019"[all fields] OR "novel coronavirus"[all fields] OR "new coronavirus"[all fields] OR "novel corona virus"[all fields] OR "new corona virus"[all fields] OR "SARS-CoV-2"[all fields] OR "SARSCoV2"[all fields] OR "SARS-CoV2"[all fields] OR "2019nCoV"[all fields] OR "2019-nCoV"[all fields] OR "2019 coronavirus"[all fields] OR "2019 corona virus"[all fields] OR "coronavirus disease 2019"[all fields] OR "severe acute respiratory syndrome coronavirus 2"[nm] OR "severe acute respiratory syndrome coronavirus 2"[all fields] OR "sars-coronavirus-2"[all fields] OR "coronavirus disease 2019"[all fields] OR "corona virus disease 2019")

AND

(("control"[Title] OR "spread*"[Title] OR "contain*"[all fields])))

AND

(((partial[all fields] OR full[all fields] OR national[all fields] OR state[all fields] OR local[all fields] OR limited[all fields] OR regional[all fields]) AND (lockdown*[all fields] OR "lock down*"[all fields] ) OR "containment measure*"[all fields] OR "stay at home"[all fields])))

AND

("opening up" [all fields] OR "open up"[all fields] OR "exit strategy*" [all fields] OR (removal*[all fields] AND (lockdown* [all fields] OR "lock down*"[all fields])) OR ((exit[all fields] OR end[all fields]) AND "stay at home"[all fields]))
